# Pathological fractures in predicting clinical outcomes for patients with osteosarcoma

**DOI:** 10.1186/s12891-016-1351-x

**Published:** 2016-12-28

**Authors:** Lien-Hsiang Chung, Po-Kuei Wu, Cheng-Fong Chen, Hung-Kai Weng, Tain-Hsiung Chen, Wei-Ming Chen

**Affiliations:** 1 Department of Orthopaedics, Therapeutical and Research Center of Musculoskeletal Tumor, Taipei Veterans General Hospital, No. 201, Sec. 2, Shih-Pai Rd., Taipei, 112 Taiwan; 2 Rehabilitation and Technical Aid Center, Taipei Veterans General Hospital, Taipei, Taiwan; 3 Department of Orthopaedics & Traumatology, Taipei Veterans General Hospital, Taipei, Taiwan; 4 Department of Orthopaedics, Chia-Yi Yang Ming Hospital, Chia-Yi, Taiwan; 5 Institute of Clinical Medicine, National Yang-Ming University, Taipei, Taiwan; 6 Department of Orthopedic Surgery, School of Medicine, National Yang-Ming University, Taipei, Taiwan; 7 Department of Orthopedic Surgery, National Cheng Kung University Hospital; College of Medicine, National Cheng Kung University, Tainan and Dou-Liou Branch, Taiwan; 8 Institute of Basic Medical Sciences, College of Medicine, National Cheng Kung University, Tainan, Taiwan

**Keywords:** Pathological fracture, Osteosarcoma, Survival, Prognostic factors, Chemotherapy, Lung metastasis

## Abstract

**Background:**

Studies reported contradictory results for the prognostic significance of a pathological fracture in osteosarcoma patients. The aim of this study is to report the outcomes for a cohort of patients with osteosarcoma who presented with and without pathological fractures and to identify the prognostic importance of pathological fracture in predicting outcomes and influences on survival.

**Methods:**

Data of patients with osteosarcoma were retrospectively reviewed. Between March 1992 and June 2014, a total of 268 patients with osteosarcoma were included in this analysis, of whom 34 (12.7%) with fractures at diagnosis or sustained after chemotherapy and 234 (87.3%) without fracture. All patients were treated with approaches that integrated chemotherapy and surgical resections to maximal extent of all sites whenever feasible. The association between potential prognostic factors and survival for these patients were analyzed and compared.

**Results:**

No significant difference was observed in overall survival, progression free survival, and disease free survival between osteosarcoma patients with pathological fractures and without fracture. The patients without fracture had a 5-year survival of 50% and 10-year survival of 21%, in contrast to 37% (5-year) and 22% (10-year) in patients with fractures. Lung metastasis was the significant predictor for the presence of fractures. Advanced stage (III) of tumor, lung metastasis, poor response to chemotherapy, and local recurrence were associated increased risk for death in all osteosarcoma patients.

**Conclusion:**

Pathological fracture is not a predictor of worse survival in this study. Further studies with matched cases are needed to confirm our observations.

## Background

Pathological fracture in osteosarcoma, with an incidence from 5% to 12%, can be the presenting feature at diagnosis of or occur during treatment [1]. Studies reported that metastases occur in 10–20% of osteosarcoma patients at the time of diagnosis [2], and lung and bone are the most common sites [2, 3]. The current multidisciplinary treatment program for osteosarcoma, typically composed of surgical management, pre- and post-operative chemotherapy, has provided about a cure rate of for patients without clinically evident metastasis at presentation [4, 5]. However, for the osteosarcoma patients present with metastatic disease, the reported 5-year survival rates ranged from 10% to 50% [3]. Significant predictors of survival for osteosarcoma patients include metastases, anatomic site, histological response to chemotherapy, serum levels of alkaline phosphatase, and lactate dehydrogenase [5].

Pathological fractures in osteosarcoma patients are considered to induce hematomas, increase the risk of unexpected micro-metastasis [6, 7] and has been linked to the higher mortality rates as a result of metastatic cancer progression [1]. The management of osteosarcoma is complicated by pathological fracture; however, studies have reported contradictory implications of pathological fracture, in which similar survival [8, 9] and worse survival [10–12] were indicated. Some recent studies reported that a pathological fracture has no significant prognostic value in patients with high-grade extremity osteosarcoma [13, 14] and is a poor predictor for local recurrence despite an association with increased mortality [15]. Nevertheless, Scully et al. concluded that pathological fractures in patients previously treated for osteosarcoma can be used as a risk factor for local recurrence [11]. Because of the relative rarity of osteosarcoma patients, most studies examining pathological fractures in osteosarcoma patients [9, 13–18] included patients might be matched for some criteria (e.g. cancer stage, localized disease), but varied widely in other important parameters (such as chemotherapy regimens, metastasis, age, lesion site, surgical extent, limb amputation or salvage, etc.). Thus, comparisons between studies have been difficult and the usefulness of pathological fracture as a prognostic indicator of survival outcome or recurrence remains controversial. According to a previous study investigating the effects of manipulative therapy on the prognosis for osteosarcoma’s patients in our institution [19], surgery followed by neoadjuvant chemotherapy could boost overall survival rate to 92%, in contrast to 58% of patients received manipulative therapy and they were associated with significantly higher rates of metastasis and poorer prognosis (*p* < 0.05). As we have identified manipulative therapy a risk factor of survival rate, this study was therefore undertaken to examine whether a pathologic fracture in patients with osteosarcoma has prognostic importance in predicting outcomes and influences on survival.

## Methods

### Screening of eligible patients

We retrospectively reviewed the medical charts of patients with osteosarcoma who were treated and followed up at Taipei General Veterans Hospital between March 1992 and June 2010. This study was conducted following the approval of Institutional Review Board of Ditmanson Medical Foundation Chia-Yi Christian Hospital (Taiwan) and the obtaining of patients’ informed consent. Data were retrieved from the medical charts, including age at diagnosis, gender, histology of osteosarcoma, anatomic location of tumor and related fracture, categorization of fracture, the presence of metastasis and time, details of therapeutic regimens, surgical therapy, responses to chemotherapy, and dates of the last follow-up or death. Patients were included in this study based on (1) presentation of osteosarcoma of the femur, humerus, tibia, fibula or other areas; (2) no previous pathological fracture. Patients excluded were those with (1) previous diagnosis of pathological fracture; (2) other cancer history or treatment, and (3) lost to follow-up or incomplete.

### Diagnosis and follow-ups

All the patients with pathologically confirmed diagnosis of osteosarcoma underwent computer tomography (CT) scan, magnetic resonance imaging (MRI), and/or sonography. The presence of a pathological fracture in these patients was also obtained from the medical charts of the whole course of treatment. Tumors were staged according to Enneking’s Musculoskeletal Tumor Society system [20]. Tumor sizes were calculated using the formula: 0.52 × [width (mm) × height (mm) × length (mm)] [21], with the measurements determined from MRI. Tissue diagnoses were obtained by needle biopsy for all the cases.

The follow-up protocols and imaging schedules were consistent during the period. CT scan of the chest was performed before surgery, every three months in the first two years post-operatively, every six months during the third to fifth years, and then annually thereafter. Local recurrences over the primary tumor location were monitored with roentgenographies, and either MRI and/or sonography on the same schedule. The imaging studies were reviewed and interpreted by certified radiologists of this institution. Both groups of patients with a pathological fracture and without fracture were observed in the outpatient clinics following discharge until either relapse or death was reported. Physical examination, radiograph, CT scan, bone scan, and MRI were included in follow-up assessments.

### Clinical management and treatment strategies

The patients were treated according the most appropriate therapeutic regimen for each patient at the time of the treatment, involving chemotherapy and surgical interventions. The pre-operative neoadjuvant chemotherapy regimen was standardized after 2003, including 12 mg/m^2^ methotrexate, 37.5 mg/m^2^/day adriamycin, 3.0 mg/m^2^/day ifosfamide and 60 mg/m^2^/day cisplatin for a minimum of 2 cycles [22]. Adjuvant chemotherapy following surgery was provided according to the guidelines of National Comprehensive Cancer Network (NCCN) for bone cancer. The response to neoadjuvant chemotherapy was evaluated by pathological estimation of the resected specimen according to the method defined by Huvos et al. [23]. A good response to chemotherapy was defined to achieve more than 90% of tumor necrosis.

After chemotherapy and reassessment, all patients received the definite tumor surgery based on their responses to chemotherapy, location and extension of tumor, and patient age, to achieve wide surgical margins as much as possible. Limb-salvage and limb-sacrificing procedures were considered according to chemotherapeutic responses or based on patient preference. All the pathological fractures were managed according to guidelines at that time and each patient needs [24], including temporally external fixation for femoral diaphysis and followed by limb salvage surgery. Distal femoral and proximal tibial metaphysis were stabilized with long leg cast through the whole course of preoperative treatment. No fixation device was applied for proximal femoral metaphysis. Ambulation with crutches was allowed for these patients whenever necessary.

### Statistical analysis

Patients were categorized into two groups according to the presence and absence of a pathological fracture. All data were analyzed using the SPSS software, version 16.0 (SPSS Inc., Chicago, IL, USA) and expressed as mean ± standard deviation (SD) for numeral data or frequencies for categorical data. A *p*-value of less than 0.05 was considered to indicate a statistically significant difference. The differences in the demographic data and the clinical characteristics between the two groups were evaluated by Pearson chi-squared tests. Comparisons of the variables such as age, gender, tumor stage and response to chemotherapy between the groups were accessed by independent Student’s *t*-test.

Cox regression model was used to conduct multivariate analysis. Variables were evaluated to determine their prognostic values in relations to overall survival (OS), progression free survival (PFS) and disease free survival (DFS). OS was calculated from the time of diagnosis until the last follow-up or death. PFS was calculated from the time of diagnosis until the first documentation of progression (metastasis) or recurrence, last follow-up or death. DFS was defined as the period of no evidence of disease survival following curative therapy (chemotherapy and surgery) until the last follow-up.

## Results

### Characteristics and outcomes

From March 1992 to June 2014, a total of 268 patients with osteosarcoma had received a complete treatment protocol without any improper intervention, including 12.7% patients (*n* = 34) with sustained pathological fractures related to osteosarcoma and 87.3% patients (*n* = 234) without fracture. Of the patients with fractures, 65% had a fracture at diagnosis and the others developed a fracture after biopsy or chemotherapy (data not shown). As summarized in Table 1, although the overall ratio of males to females was 1.48 (*n* = 160/108), female patients had significantly higher frequency of fractures (*p* = 0.030). Mean ages at diagnosis were 22.8 ± 15.2 and 23.5 ± 19.1 years, and no difference in the categorized age groups. The majority of patients presented with Enneking stage-IIB disease, including 86.3% (*n* = 202) patients with fractures and 79.4% (*n* = 29) patients without fractures. No significant difference was observed in the distribution of tumor stages (*p* = 0.112) and mean sizes of tumor (*p* = 0.907) between patients with and without fractures. Femur, tibia, and humerus accounted for more than 70% of tumors. The patients with fractures had higher percentages of tumors presented at femur and humerus, but a lower frequency occurred at tibia when compared with those without fracture. A significant higher percentage of lung metastasis, either at initial presentation or occurred during follow-up, was found in the patients with a fracture (50.0% vs. 32.1%; *p* = 0.039). No significant difference was detected for the rates of local recurrence or average duration to recurrence following treatment.

**Table 1:** Demographics, disease-related characteristics and outcomes of non-fracture and fracture groups in 268 patients with osteosarcoma

Characteristics	Non- Fracture *N* = 234 (87.3%)		Fracture *N* = 34 (12.7%)		p*
	*N*	%, or mean ± SD	*N*	%, or mean ± SD	
Gender					
Males	141	60.3	19	55.9	0.030
Females	93	39.7	15	44.1	
Age, years					
Mean		22.8 ± 15.2		23.5 ± 19.1	0.836
≤ 10	24	10.3	5	14.7	0.626
10–20	124	53.0	19	55.9	
> 20	84	35.9	10	29.4	
Stage of tumor ^a^					
IB	4	3.2	0	0	0.112
IIB	202	86.3	27	79.4	
III	28	22.6	7	38.9	
Tumor size, mm^2^	148	363.7 ± 641.1	16	389.4 ± 845.9	0.907
Tumor location					
Femur	94	40.2	18	52.9	0.020
Tibia	49	20.9	3	8.8	
Humerus	16	6.8	10	29.4	
Fibula	6	2.6	1	2.9	
Others ^b^	21	9.0	2	5.9	
Not Specified	48	20.5	0	0.0	
Lung metastasis ^c^					
No	159	67.9	17	50.0	0.039
Yes	75	32.1	17	50.0	
Necrosis rate, %					
< 90% (poor)	41	31.1	13	44.8	0.351
≥ 90% (good)	65	49.2	12	41.4	
No preoperative C/T ^d^	26	19.7	4	13.8	
Local recurrence					
No	183	78.2	26	76.5	0.820
Yes	51	21.8	8	23.5	
Status until last follow up					
No evidence of disease	108	46.4	16	47.1	0.904
Alive with disease	35	15.1	6	17.6	
Died of disease	89	38.4	12	35.3	
Duration to recurrence, months	41	23.1 ± 20.9	5	12.8 ± 9.4	0.084
Follow up duration, months	234	68.0 ± 52.1	34	65.2 ± 57.7	0.362
Overall survival, months	234	65.7 ± 48.9	34	56.4 ± 52.3	0.338
Progression free survival, months	134	67.9 ± 52.0	20	65.2 ± 57.7	0.841
Disease-free survival, months	108	87.1 ± 48.1	16	80.8 ± 55.1	0.667

^a^Enneking stage

^b^Others included radius, pelvis, hip, sinonasal, skull, spine, neck, and scapula

^c^ Metastasis at diagnosis or developed during follow-up

^d^C/T: chemotherapy

*Comparisons were based on the independent student *t*-test and Pearson chi square test

Similar outcomes in terms of survival were noted, approximately 46% were freed of disease while 36% eventually died as a result of tumor and 16% is still alive with the disease. Furthermore, no significant difference was observed in the responses to chemotherapy according to the degree of tissue necrosis rates and the follow-up duration [68.0 ± 52.1 (median 52.0; range 0.7–277.7) and 65.2 ± 57.7 (median 45.0; range 1.0–200.4) months, *p* = 0.362]. Survival and disease status (no evidence of disease, alive with disease, and died of disease), OS (*p* = 0.338), PFS (*p* = 0.841), and DFS (*p* = 0.667) all revealed no statistically significant difference.

### Analyses of prognostic factors

In Table 2, univariate analysis showed that only the presence of lung metastasis was a significant predictor of fracture (OR = 2.12; *p* = 0.043), but gender, poor necrosis rate (<90%), and local recurrence were not significantly correlated with the presence of fractures. Table 3 shows the analysis of variables in predicting death in osteosarcoma patients. Tumor stage III (OR = 12.00; *p* = 0.043), poor necrosis (OR = 2.11; *p* = 0.048), lung metastasis (OR = 3.32; *p* = 0.000), and local recurrence (OR = 1.81; *p* = 0.046) were significant predictors of death in patients with osteosarcoma. However, pathological fracture, age, gender, tumor stage IIB, and tumor location were not significantly associated with the survival for these patients.

**Table 2:** Odds ratios of variables associated the presence of pathological fractures in patients with osteosarcoma, by univariate logistic regression

Factor	Odds ratio	95% CI	*p* (chi-square)
Gender (female vs. male)	1.20	0.58–2.47	0.627
Poor necrosis rate (<90%)	1.72	0.72–4.13	0.227
Lung metastasis	2.12	1.03–4.38	0.043
Local recurrence	1.10	0.47–2.59	0.820

CI: confidential interval

**Table 3:** Univariate logistic regression to identify risk factors for the prediction of death in all patients with osteosarcoma (total *n* = 268)

Variable	OR	95% CI	*p*
Fracture (reference: no fracture)	0.88	0.41–1.86	0.731
Age group (reference: ≤ 10)			
10–20	1.52	0.63–3.67	0.355
>20	2.03	0.82–5.05	0.127
Gender (reference: male)	0.80	0.48–1.34	0.420
Tumor stage (reference: IB)			
IIB	1.68	0.17–16.75	0.658
III	12.00	1.08–133.61	0.043
Tumor location (reference: fibula)			
Femur	0.96	0.21–4.51	0.963
Tibia	0.52	0.12–2.96	0.524
Humerus	0.31	0.07–2.31	0.306
Necrosis rate < 90% (reference: ≥ 90%)	2.11	1.01–4.44	0.048
Lung metastasis (reference: no)	3.32	1.96–5.62	0.000
Local recurrence (reference: no)	1.81	1.01–3.25	0.046

All these findings suggest that lung metastasis appears to be a predictor of pathological fractures and a prognostic factor of survival in osteosarcoma patients with fractures. In contrast, additional logistic regression analysis demonstrated that pathological fracture is not a significant predictor of lung metastasis (OR = 0.947, 95%CI, 0.449–1.997; *p* = 0.886). In addition to lung metastasis, necrosis rate in response to chemotherapy and local recurrence were significantly associated with inferior survival in all osteosarcoma patients.

### Survival outcomes

**Fig. 1:**
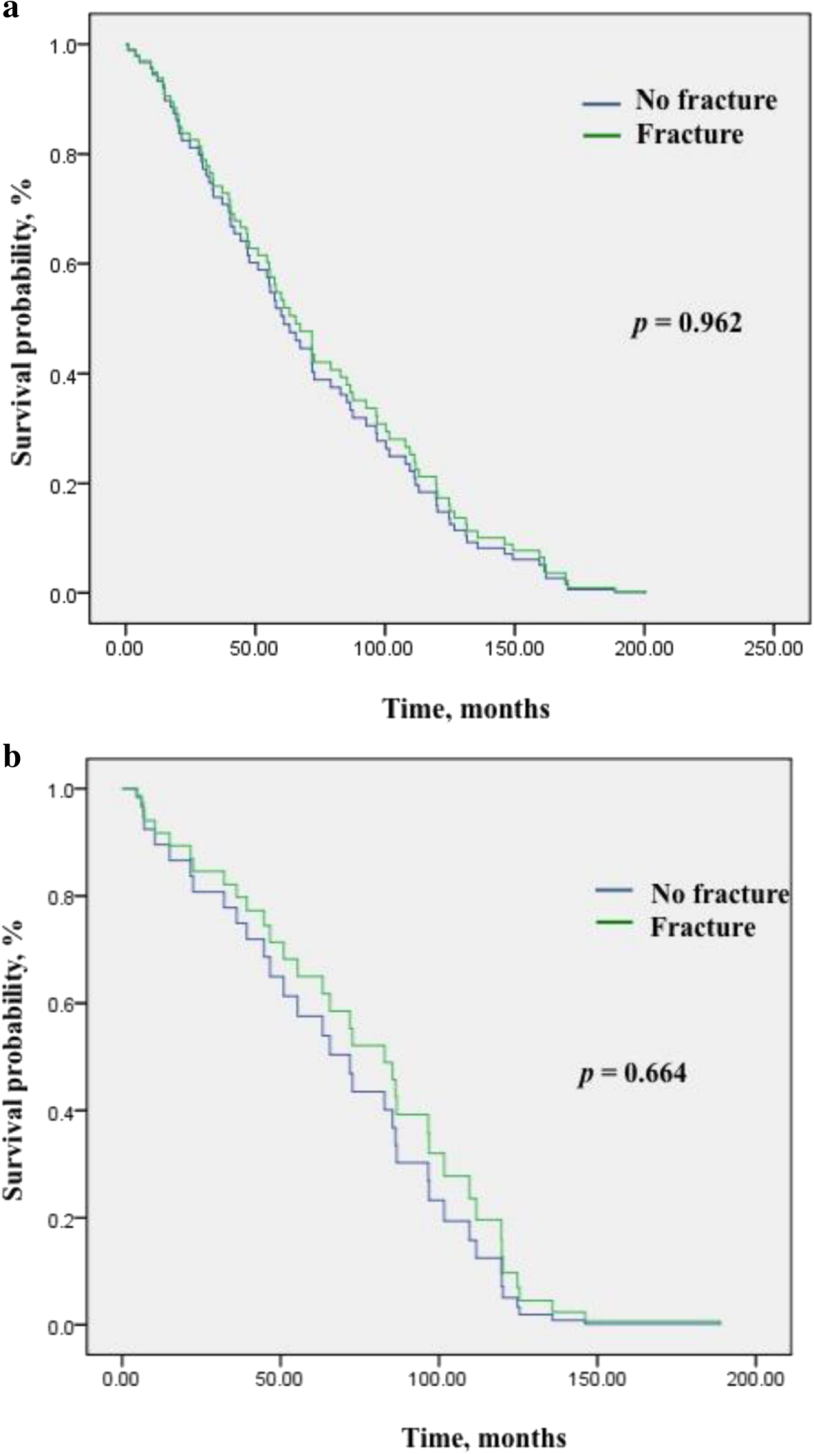
Cox regression multivariate analysis of comparing survival between osteosarcoma patients with pathological fractures and those without fracture, with simultaneous adjustment for tumor stage, metastasis, tumor size, necrosis rate, and age. No significant difference was found for **a** Overall survival (*p* = 0.962) and **b** Progression free survival (*p* = 0.664)

**Fig. 2:**
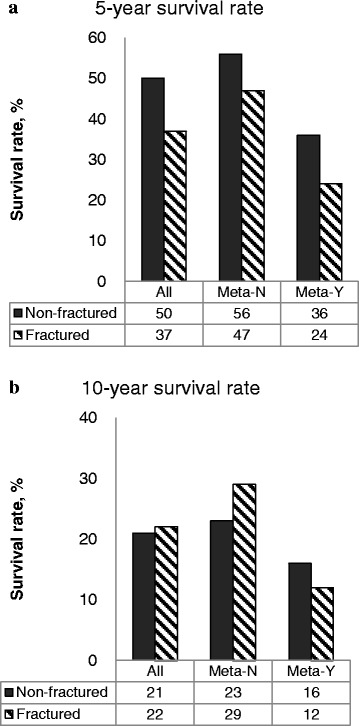
The 5-year and 10-year survival rates by fracture and lung metastasis for osteosarcoma patients. Survival outcomes varied depending on the presence of lung metastasis. Meta-N: without lung metastasis; Meta-Y: with metastases; PFS: progression free survival

The probabilities of OS and PFS by months of the whole groups are shown in Fig. 1. No significant difference was observed between the osteosarcoma patients with fractures and without fracture (*p* = 0.962 for OS, *p* = 0.664 for PFS). The 5-year and 10-year survival rates were 50% and 21% for patients without fracture compared to 37% and 22% in patients with a pathological fracture. The difference was not statistically significant (*p* = 0.312) (Fig. 2). For non-metastatic patients, the rates were 56% and 23% for those without fracture vs. 47% and 29% for those with fractures; and for metastatic patients, the rates were 36% and 16% (patients without fracture) vs. 24% and 12% (patients with fractures) (Fig. 2a & b; *p* = 0.004). Similarly, PFS rates did not significantly differ between the two whole groups, but varied greatly depending on the presence of lung metastasis, with no PFS survivor in the group of patients with fractures at the 5th year and significantly less 5-year survivors with lung metastases in patients with fractures (Fig. 2c; *p* = 0.000).

## Discussion

The present study examined the prognostic importance of pathologic fractures for osteosarcoma patients. The patients of both groups were comparable in age, tumor stage and size, local recurrence, and necrosis rate. Our results revealed no significant difference in the OS, PFS, 5-year, and 10-year survival between the two groups. The findings are similar to previous studies indicating that pathological fractures in osteosarcoma do not adversely affect survival [9, 13], unless the presence of lung metastasis. Furthermore, our study also supports the contention that proper evaluation of preoperative imaging, following by appropriate chemotherapy regimens, and surgical approaches can result in no difference in tumor necrosis rates or the risk of local recurrence between patients with fractures and without fracture, suggesting that an effective multidisciplinary team can provide consistent outcomes in spite of the management of fractured patients is more difficult.

In this study, lung metastasis was a significant predictor for fracture and one of significant risk factors for death for the entire study population. Comparing to the incidence rates of lung metastasis in osteosarcoma patients with fractures in previous studies, 19.3% [17] or 23% [11], this study clearly demonstrated a much higher proportion of patients with fractures had lung metastases, with 50% in patients with fractures and 32% in those without fracture. Although a pathological fracture *per se* was not a significant predictor for survival, lung metastases and fractures could promote the progression of osteosarcoma and led to an inferior survival in fractured patients. Metastasis at diagnosis is the only widely accepted prognostic factor [2]. The contemporary treatment regimens can result in up to 70% survival for patients with localized osteosarcoma of the extremity. But the survival estimates for patients with metastatic disease were much worse, ranging from 8.3% for 5-year [25], 23% for 5-year [26], 30% for 4-year [27], 53.3% for 5-year [28], to 55% for 2-year [29]. However, these studies varied widely in many variables, making comparisons impossible. The long-term survival data were neither reported.

Local recurrence can be a result of poor response to chemotherapy or inadequate surgical margins [30, 31]. The rates of local recurrence following osteosarcoma surgery generally ranged 4–10% [32–34]. In our study, the rates were relatively higher (21.8% and 23.5%), but lower than a more recent study reporting the rates in patients with adequate (30.5%) and inadequate (38%) surgical margins, and in which, 15% patients had pathological fractures [35]. Consistent with previous observations [32, 35, 36], local recurrence was correlated with poor prognosis in terms of survival in the present study. To determine the impact of surgical margin on the development of local recurrence, further investigation remains necessary.

Tumor size has been considered as an important risk factor for osteosarcoma patients [15] and a potential confounding factor associated with poor prognosis for those with fractures [13, 37]. Increased tumor volume might result in poor response to chemotherapy [38]; however, other studies did not find an influence of tumor size [11]. In our series, tumor size was not significantly correlated with OS for all patients, patients with or without fractures (data not shown). Moreover, we found that fractured female patients had a better survival than male counterparts in exploratory subgroup analyses, which has never been reported. We also analyzed the 10-year survival rates that were generally lack in most studies. Although the data of this study was relevant to both pediatric and adult patients, the sample size remained small so that the exploratory analyses for subgroups were limited.

## Conclusions

A pathological fracture in patients with osteosarcoma did not increase the risk of death. No significant difference was observed in OS, PFS, or DFS between osteosarcoma patients with fractures and without fracture. Lung metastasis at diagnosis was a significant predictor for the presence of a pathological fracture. Advanced stage (III) of tumor, lung metastasis, poor response to chemotherapy, and local recurrence were associated an increased risk for death in all patients with osteosarcoma. Further confirmation of the effects of a pathological fracture by comparing with case matched studies is required.

## Abbreviations

CT: Computer tomography
MRI: Magnetic resonance imaging
OS: Overall survival
PFS: Progression free survival
DFS: Disease free survival
OR: Odds ratio
SD: Standard deviation

## References

[1] Chandrasekar CR, Grimer RJ, Carter SR, Tillman RM, Abudu A, Jeys LM (2012). Pathological fracture of the proximal femur in osteosarcoma: need for early radical surgery?. ISRN Oncol.

[2] Bielack SS, Kempf-Bielack B, Delling G, Exner GU, Flege S, Helmke K (2002). Prognostic factors in high-grade osteosarcoma of the extremities or trunk: an analysis of 1,702 patients treated on neoadjuvant cooperative osteosarcoma study group protocols. J Clin Oncol.

[3] Kager L, Zoubek A, Potschger U, Kastner U, Flege S, Kempf-Bielack B (2003). Primary metastatic osteosarcoma: presentation and outcome of patients treated on neoadjuvant Cooperative Osteosarcoma Study Group protocols. J Clin Oncol.

[4] Federman N, Bernthal N, Eilber FC, Tap WD (2009). The multidisciplinary management of osteosarcoma. Curr Treat Options Oncol.

[5] Picci P (2007). Osteosarcoma (osteogenic sarcoma). Orphanet J Rare Dis.

[6] Finn HA, Simon MA (1989). Staging systems for musculoskeletal neoplasms. Orthopedics.

[7] Simon MA (1988). Limb salvage for osteosarcoma. J Bone Joint Surg Am.

[8] Abudu A, Sferopoulos NK, Tillman RM, Carter SR, Grimer RJ (1996). The surgical treatment and outcome of pathological fractures in localised osteosarcoma. J Bone Joint Surg (Br).

[9] Bacci G, Ferrari S, Longhi A, Donati D, Manfrini M, Giacomini S (2003). Nonmetastatic osteosarcoma of the extremity with pathologic fracture at presentation: local and systemic control by amputation or limb salvage after preoperative chemotherapy. Acta Orthop Scand.

[10] Glasser DB, Lane JM, Huvos AG, Marcove RC, Rosen G (1992). Survival, prognosis, and therapeutic response in osteogenic sarcoma. The Memorial Hospital experience Cancer.

[11] Scully SP, Ghert MA, Zurakowski D, Thompson RC, Gebhardt MC (2002). Pathologic fracture in osteosarcoma : prognostic importance and treatment implications. J Bone Joint Surg Am.

[12] Vermesan D, Vermesan H, Dragulescu SI, Bera I, Di Giovanni A, Sabatini R (2009). Secondary pathologic fractures in osteosarcoma: prognosis and evolution. Eur Rev Med Pharmacol Sci.

[13] Kim MS, Lee SY, Lee TR, Cho WH, Song WS, Cho SH (2009). Prognostic effect of pathologic fracture in localized osteosarcoma: a cohort/case controlled study at a single institute. J Surg Oncol.

[14] Xie L, Guo W, Li Y, Ji T, Sun X (2012). Pathologic fracture does not influence local recurrence and survival in high-grade extremity osteosarcoma with adequate surgical margins. J Surg Oncol.

[15] Ferguson PC, McLaughlin CE, Griffin AM, Bell RS, Deheshi BM, Wunder JS (2010). Clinical and functional outcomes of patients with a pathologic fracture in high-grade osteosarcoma. J Surg Oncol.

[16] Bramer JA, Abudu AA, Grimer RJ, Carter SR, Tillman RM (2007). Do pathological fractures influence survival and local recurrence rate in bony sarcomas?. Eur J Cancer.

[17] Ebeid W, Amin S, Abdelmegid A (2005). Limb salvage management of pathologic fractures of primary malignant bone tumors. Cancer Control.

[18] Natarajan MV, Govardhan RH, Williams S, Raja Gopal TS (2000). Limb salvage surgery for pathological fractures in osteosarcoma. Int Orthop.

[19] Wu PK, Chen WM, Lee OK, Chen CF, Huang CK, Chen TH (2010). The prognosis for patients with osteosarcoma who have received prior manipulative therapy. J Bone Joint Surg (Br).

[20] Enneking WF, Spanier SS, Goodman MA (1980). A system for the surgical staging of musculoskeletal sarcoma. Clin Orthop Relat Res.

[21] Li M, Zhang Y, Liu Z, Bharadwaj U, Wang H, Wang X (2007). Aberrant expression of zinc transporter ZIP4 (SLC39A4) significantly contributes to human pancreatic cancer pathogenesis and progression. Proc Natl Acad Sci U S A.

[22] Bacci G, Briccoli A, Rocca M, Ferrari S, Donati D, Longhi A (2003). Neoadjuvant chemotherapy for osteosarcoma of the extremities with metastases at presentation: recent experience at the Rizzoli Institute in 57 patients treated with cisplatin, doxorubicin, and a high dose of methotrexate and ifosfamide. Ann Oncol.

[23] Huvos AG, Rosen G, Marcove RC (1977). Primary osteogenic sarcoma: pathologic aspects in 20 patients after treatment with chemotherapy en bloc resection, and prosthetic bone replacement. Arch Pathol Lab Med.

[24] Bacci G (2003). Pathologic fracture in osteosarcoma. J Bone Joint Surg Am.

[25] Daw NC, Billups CA, Rodriguez-Galindo C, McCarville MB, Rao BN, Cain AM (2006). Metastatic osteosarcoma. Cancer.

[26] Salah S, Ahmad R, Sultan I, Yaser S, Shehadeh A (2014). Osteosarcoma with metastasis at initial diagnosis: Current outcomes and prognostic factors in the context of a comprehensive cancer center. Mol Clin Oncol.

[27] Marina NM, Pratt CB, Rao BN, Shema SJ, Meyer WH (1992). Improved prognosis of children with osteosarcoma metastatic to the lung(s) at the time of diagnosis. Cancer.

[28] Harris MB, Gieser P, Goorin AM, Ayala A, Shochat SJ, Ferguson WS (1998). Treatment of metastatic osteosarcoma at diagnosis: a Pediatric Oncology Group Study. J Clin Oncol.

[29] Goorin AM, Harris MB, Bernstein M, Ferguson W, Devidas M, Siegal GP (2002). Phase II/III trial of etoposide and high-dose ifosfamide in newly diagnosed metastatic osteosarcoma: a pediatric oncology group trial. J Clin Oncol.

[30] Picci P, Sangiorgi L, Rougraff BT, Neff JR, Casadei R, Campanacci M (1994). Relationship of chemotherapy-induced necrosis and surgical margins to local recurrence in osteosarcoma. J Clin Oncol.

[31] Picci P, Sangiorgi L, Bahamonde L, Aluigi P, Bibiloni J, Zavatta M (1997). Risk factors for local recurrences after limb-salvage surgery for high-grade osteosarcoma of the extremities. Ann Oncol.

[32] Bacci G, Longhi A, Cesari M, Versari M, Bertoni F (2006). Influence of local recurrence on survival in patients with extremity osteosarcoma treated with neoadjuvant chemotherapy: the experience of a single institution with 44 patients. Cancer.

[33] Kempf-Bielack B, Bielack SS, Jurgens H, Branscheid D, Berdel WE, Exner GU (2005). Osteosarcoma relapse after combined modality therapy: an analysis of unselected patients in the Cooperative Osteosarcoma Study Group (COSS). J Clin Oncol.

[34] Nathan SS, Gorlick R, Bukata S, Chou A, Morris CD, Boland PJ (2006). Treatment algorithm for locally recurrent osteosarcoma based on local disease-free interval and the presence of lung metastasis. Cancer.

[35] Kong CB, Song WS, Cho WH, Oh JM, Jeon DG (2012). Local recurrence has only a small effect on survival in high-risk extremity osteosarcoma. Clin Orthop Relat Res.

[36] Bacci G, Forni C, Longhi A, Ferrari S, Mercuri M, Bertoni F (2007). Local recurrence and local control of non-metastatic osteosarcoma of the extremities: a 27-year experience in a single institution. J Surg Oncol.

[37] Bacci G, Longhi A, Versari M, Mercuri M, Briccoli A, Picci P (2006). Prognostic factors for osteosarcoma of the extremity treated with neoadjuvant chemotherapy: 15-year experience in 789 patients treated at a single institution. Cancer.

[38] Cho WH, Song WS, Jeon DG, Kong CB, Kim MS, Lee JA (2010). Differential presentations, clinical courses, and survivals of osteosarcomas of the proximal humerus over other extremity locations. Ann Surg Oncol.

